# Morphological differences of the neuroretinal rim between temporally tilted and non-tilted optic discs in healthy eyes

**DOI:** 10.1038/s41598-024-54116-7

**Published:** 2024-03-13

**Authors:** Chan Woong Joo, Youn Joo Choi, Han Ul Kim, Sung Pyo Park, Kyeong Ik Na

**Affiliations:** 1 Department of Ophthalmology, Kangdong Sacred Heart Hospital, Hallym University College of Medicine, #150 Seongan-ro, Gangdong-gu, Seoul, 05355 South Korea; 2 Department of Ophthalmology, Armed Forces Seoul District Hospital, Seoul, South Korea

**Keywords:** Diseases, Medical research

## Abstract

This study aimed to compare morphological differences of the neuroretinal rim between the temporally tilted and non-tilted optic discs in healthy eyes. We prospectively enrolled participants aged 20–40 years with temporally tilted or non-tilted optic discs. The optic nerve head parameters were analyzed using spectral domain-optical coherence tomography. The angle between the Bruch’s membrane opening (BMO) plane and BMO-minimum rim width (BMO-MRW) was termed “BMO-MRW angle”. Peripapillary retinal nerve fiber layer thickness (pRNFLT) and BMO-based parameters were compared between the temporally tilted and non-tilted disc groups. As a result, 55 temporally tilted disc eyes and 38 non-tilted disc eyes were analyzed. Global pRNFLT, global BMO-MRW, and total BMO-minimum rim area (BMO-MRA) were similar between the two groups (*p* = 0.138, 0.161, and *p* = 0.410, respectively). In the sectoral analysis, temporally tilted disc group exhibited thicker BMO-MRW in the temporal sector (*p* = 0.032) and thinner in the nasal superior and nasal sectors (*p* = 0.025 and *p* = 0.002, respectively). Temporally tilted disc group showed larger BMO-MRA in the temporal, temporal superior, and temporal inferior sectors (*p* < 0.001, *p* < 0.001, and *p* < 0.016, respectively), alongside a higher BMO-MRW angle in the temporal sector and lower in the nasal superior and nasal sectors. In conclusion, the neuroretinal rim, represented by BMO-MRW and BMO-MRA, showed morphological differences between temporally tilted and non-tilted optic discs in healthy eyes. BMO-MRW and BMO-MRA showed temporalization in the same manner as pRNFLT in the temporally tilted disc eyes. The BMO-MRW angle showed that in temporally tilted disc eyes, optic nerve fibers met the BMO plane steeply in the nasal sector and gently in the temporal sector than in non-tilted disc eyes, suggesting potential stress region of optic nerve fibers in temporally tilted disc eyes.

## Introduction

Glaucoma is a degenerative optic neuropathy characterized by retinal ganglion cell loss, which leads to changes in the retinal nerve fiber layer (RNFL) and optic nerve head (ONH) structure. Structural alterations of the ONH, including neuroretinal rim thinning, optic cup enlargement, and RNFL loss, are important clinical indicators of glaucoma progression and severity^1,2^.

Myopia is currently a major concern worldwide, especially in some East Asian countries due to its high prevalence and increased severity^3^. It is considered one of the risk factors for glaucoma development owing to its contribution to axial elongation and associated anatomical changes such as posterior scleral remodeling^4–6^. Myopic tilted optic disc, which is a prevalent acquired change associated with myopic progression, usually manifests as a temporal tilt of the optic disc^7–10^. This can cause structural deformation of the optic nerve, including oblique insertion, a large temporal peripapillary crescent, and temporalization of the RNFL making the diagnosis and management of glaucoma challenging in myopic eyes. Therefore, it is important to accurately evaluate the structural changes of the ONH in myopic eyes with tilted optic disc, particularly in the context of glaucoma diagnosis and management^4,11^.

Many studies have investigated the implications of a myopic tilted optic disc on the neuroretinal rim analysis in glaucomatous eyes^12–18^. In addition, several studies have explored the influence of myopia and a tilted optic disc on morphological changes of the ONH structure in healthy eyes^19–26^. Meanwhile, Bruch’s membrane opening (BMO)-based parameters in spectral domain-optical coherence tomography (SD-OCT), the new structural measurements for analyzing the ONH, have become widely used diagnostic aids in glaucoma^27,28^. To the best of our knowledge, no previous reports have assessed the BMO-based parameters such as BMO-minimum rim width (BMO-MRW) and BMO-minimum rim area (BMO-MRA), regarding the optic disc tilt in healthy eyes.

The aim of this study was to investigate morphological differences of the neuroretinal rim, including BMO-MRW and BMO-MRA, between eyes with temporally tilted and non-tilted optic discs in healthy individuals. Additionally, we aimed to compare other parameters, such as peripapillary retinal nerve fiber layer thickness (pRNFLT), and the angle between the BMO plane and BMO-MRW, which we termed as the “BMO-MRW angle”.

## Results

In this study, a total of 93 eyes (55 with temporally tilted discs and 38 with non-tilted discs) from 93 subjects were included. There were no differences regarding of age (*p* = 0.560), gender (*p* = 0.184), laterality (*p* = 0.737), and laser refractive surgery (*p* = 0.120) between the temporally tilted and non-tilted disc groups. Additionally, the best-corrected visual acuity (BCVA), and intraocular pressure were similar between the two groups (*p* = 0.463 and *p* = 0.976, respectively). The visual field parameters were similar between the two groups. However, the temporally tilted disc group exhibited a lower spherical equivalent than the non-tilted disc group, with borderline significance (*p* = 0.076). The temporally tilted disc group showed a thinner central corneal thickness and longer axial length compared to the non-tilted disc group (*p* = 0.031 and *p* = 0.001, respectively). In addition, the ovality index was significantly lower in the temporally tilted disc group than in the non-tilted disc group (*p* < 0.001) (Table 1).

**Table 1:** Comparison of demographic and clinical characteristics between the temporally tilted and non-tilted disc groups.

	All subjects (n = 93)	Temporally tilted disc group (n = 55)	Non-tilted disc group (n = 38)	*p*-value
Age (years)	31.03 ± 5.47	31.31 ± 5.72	30.63 ± 5.13	0.560*
Male, n (%)	51 (54.8)	27 (49.1)	24 (63.2)	0.184^†^
Laterality (right/left)	46/47	28/27	18/20	0.737^†^
Laser refractive surgery, n (%)	33 (35.5)	22 (40.0)	9 (23.7)	0.120^†^
BCVA (logMAR)	0.03 ± 0.08	0.03 ± 0.06	0.04 ± 0.09	0.463*
Intraocular pressure (mmHg)	12.59 ± 3.62	12.58 ± 3.78	12.61 ± 3.43	0.976*
Spherical equivalent (diopter)	− 3.15 ± 3.15	− 3.63 ± 3.49	− 2.45 ± 2.44	0.076*
Central corneal thickness (μm)	507.61 ± 46.45	497.20 ± 47.94	520.55 ± 41.69	0.031*
Axial length (mm)	25.72 ± 1.42	26.21 ± 1.02	25.11 ± 1.61	0.001*
Ovality index	0.83 ± 0.09	0.75 ± 0.04	0.93 ± 0.03	< 0.001*
Visual field parameters				
SAP VFI (%)	97.58 ± 9.69	98.61 ± 1.76	96.11 ± 14.92	0.309*
SAP mean deviation (dB)	− 1.78 ± 1.62	− 1.78 ± 1.65	− 1.79 ± 1.61	0.963*
SAP pattern standard deviation (dB)	1.80 ± 0.81	1.83 ± 0.84	1.77 ± 0.79	0.702*

Values are presented as mean ± standard deviation unless otherwise indicated.

*BCVA* best-corrected visual acuity; *logMAR* logarithm of the minimum angle of resolution; *SAP* standard automated perimetry; *VFI* visual field index.

*P*-values are for comparison of the temporally tilted and non-tilted disc groups based on *Student’s *t*-test and ^†^χ^2^ test.

**Figure 1:**
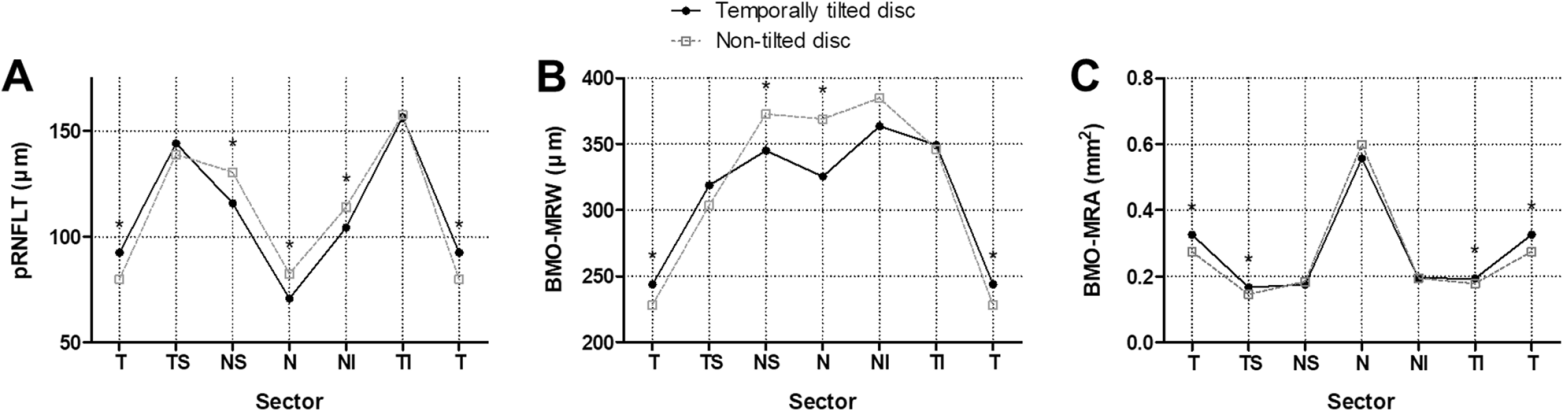
Sectoral comparison of (**A**) peripapillary retinal nerve fiber layer thickness, (**B**) Bruch’s membrane opening-minimum rim width, and (**C**) Bruch’s membrane opening-minimum rim area between temporally tilted and non-tilted disc groups. Abbreviations: pRNFLT, peripapillary retinal nerve fiber layer thickness; BMO-MRW, Bruch’s membrane opening-minimum rim width; BMO-MRA, Bruch’s membrane opening-minimum rim area; T, temporal; TS, temporal superior; NS, nasal superior; N, nasal; NI, nasal inferior; TI, temporal inferior. *P-value for comparison of the two groups based on Student’s *t*-test < 0.05.

The pRNFLT, BMO-MRW, BMO-MRA and BMO-MRW angle were compared globally and in each sector between the two groups. Global pRNFLT was similar between the two groups (*p* = 0.138). However, the temporally tilted disc group showed thicker pRNFLT in the temporal sector (*p* < 0.001) and thinner pRNFLT in the nasal superior, nasal, and nasal inferior sectors compared to the non-tilted disc group (*p* = 0.002, *p* < 0.001, and *p* = 0.013, respectively) (Table 2, Fig. 1). In the comparison of pRNFLT between the temporal and nasal sectors in each temporally tilted disc group and non-tilted disc group, pRNFLT in the temporal sector was significantly thicker than that in the nasal sector in the temporally tilted disc group (92.60 ± 18.67 vs 70.73 ± 15.18, *p* < 0.001). However, in the non-tilted disc group, pRNFLT in the temporal sector was slightly thinner than that in the nasal sector without a significant difference (79.82 ± 11.56 vs 82.45 ± 12.64, *p* = 0.347).

**Table 2:** Comparison of peripapillary retinal nerve fiber layer thickness, and optic nerve head parameters between the temporally tilted and non-tilted disc groups.

	All subjects (n = 93)	Temporally tilted disc group (n = 55)	Non-tilted disc group (n = 38)	*p*-value
pRNFLT				
Global pRNFLT (μm)	103.61 ± 7.77	102.62 ± 8.30	105.05 ± 6.77	0.138
Temporal pRNFLT (μm)	87.38 ± 17.27	92.60 ± 18.67	79.82 ± 11.56	< 0.001
Temporal Superior pRNFLT (μm)	141.96 ± 19.80	144.07 ± 20.29	138.89 ± 18.93	0.217
Nasal Superior pRNFLT (μm)	121.75 ± 23.03	115.76 ± 23.23	130.42 ± 20.02	0.002
Nasal pRNFLT (μm)	75.52 ± 15.27	70.73 ± 15.18	82.45 ± 12.64	< 0.001
Nasal Inferior pRNFLT (μm)	108.27 ± 18.14	104.40 ± 16.48	113.87 ± 19.16	0.013
Temporal Inferior pRNFLT (μm)	156.92 ± 16.97	156.53 ± 19.68	157.50 ± 12.24	0.770
BMO Area (mm^2^)	2.39 ± 0.43	2.53 ± 0.44	2.20 ± 0.33	< 0.001
BMO-MRW				
Global BMO-MRW (μm)	317.61 ± 53.39	311.73 ± 62.57	326.13 ± 35.31	0.161
Temporal BMO-MRW (μm)	237.44 ± 34.86	243.85 ± 37.62	228.16 ± 28.42	0.032
Temporal Superior BMO-MRW (μm)	312.62 ± 45.03	318.82 ± 46.10	303.66 ± 42.43	0.111
Nasal Superior BMO-MRW (μm)	356.38 ± 58.66	345.07 ± 62.14	372.74 ± 49.55	0.025
Nasal BMO-MRW (μm)	343.28 ± 71.93	325.49 ± 78.18	369.03 ± 52.84	0.002
Nasal Inferior BMO-MRW (μm)	372.26 ± 57.37	363.64 ± 65.32	384.74 ± 41.07	0.059
Temporal Inferior BMO-MRW (μm)	348.06 ± 50.73	349.27 ± 56.75	346.32 ± 41.16	0.784
BMO-MRA				
Total BMO-MRA (mm^2^)	1.602 ± 0.237	1.617 ± 0.277	1.579 ± 0.165	0.410
Temporal BMO-MRA (mm^2^)	0.306 ± 0.057	0.327 ± 0.054	0.276 ± 0.047	< 0.001
Temporal Superior BMO-MRA (mm^2^)	0.159 ± 0.024	0.168 ± 0.024	0.146 ± 0.019	< 0.001
Nasal Superior BMO-MRA (mm^2^)	0.180 ± 0.028	0.176 ± 0.031	0.185 ± 0.023	0.140
Nasal BMO-MRA (mm^2^)	0.575 ± 0.114	0.557 ± 0.129	0.599 ± 0.082	0.059
Nasal Inferior BMO-MRA (mm^2^)	0.195 ± 0.035	0.195 ± 0.040	0.195 ± 0.026	0.978
Temporal Inferior BMO-MRA (mm^2^)	0.187 ± 0.237	0.194 ± 0.039	0.178 ± 0.022	0.016

Values are presented as mean ± standard deviation unless otherwise indicated.

Abbreviations: pRNFLT, peripapillary retinal nerve fiber layer thickness; BMO-MRW, Bruch’s membrane opening-minimum rim width; BMO-MRA, Bruch’s membrane opening-minimum rim area.

*P*-values are for comparison of the temporally tilted and non-tilted disc groups based on Student’s *t*-test for all variables.

**Figure 2:**
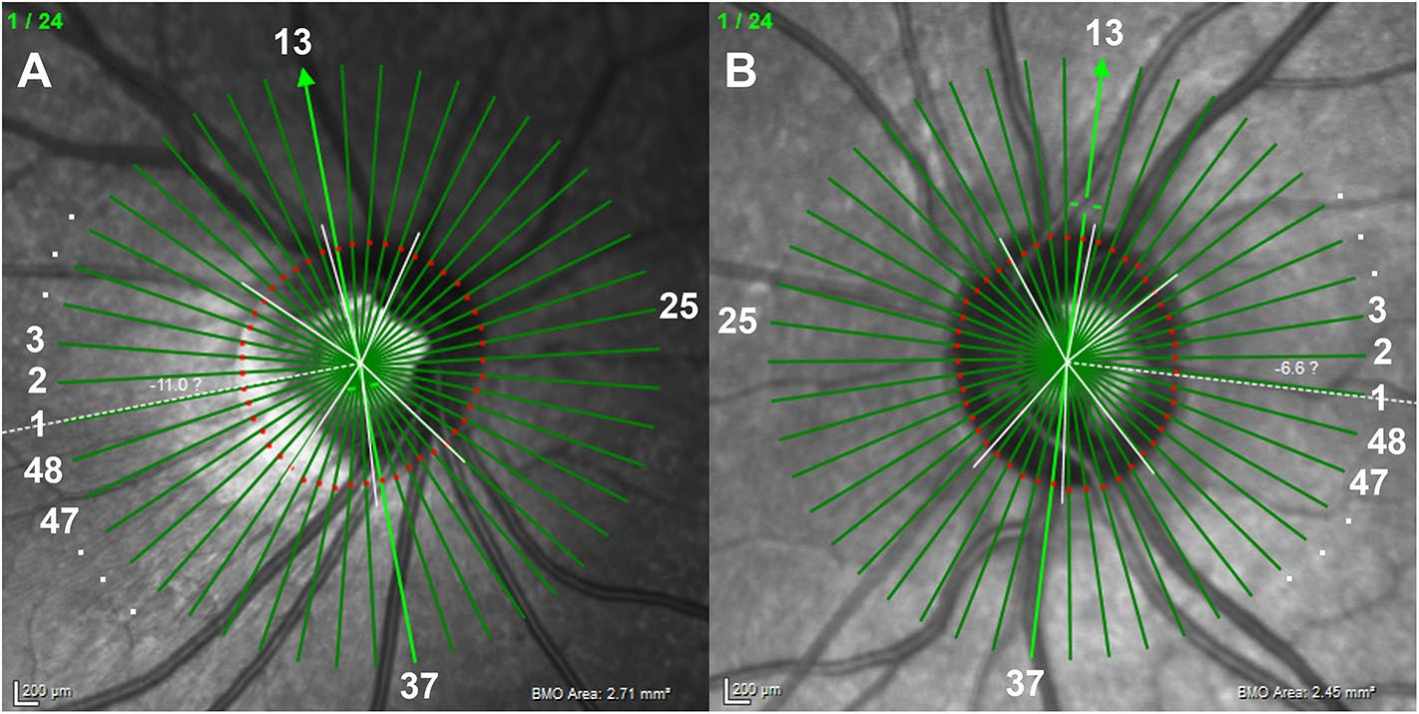
Representative infrared spectral domain-optical coherence tomography (SD-OCT) scans in eyes of temporally tilted and non-tilted disc groups. (**A**) An infrared SD-OCT scan of the right eye from a subject in the temporally tilted disc group. (**B**) An infrared SD-OCT scan of the left eye from a subject in the non-tilted disc group. A total of 24 Bruch’s membrane opening (BMO) planes and 48 different neuroretinal rim segments using a circular sector of 7.5°; number 1 indicated in white refers to the first rim segment, which is aligned parallel to the fovea-BMO center axis. Successively, the rim segment lines are numbered from 1 to 48 beginning from the temporal region, moving sequentially through superior, nasal, and inferior regions for each eye. Abbreviations: BMO, Bruch’s membrane opening.

**Figure 3:**
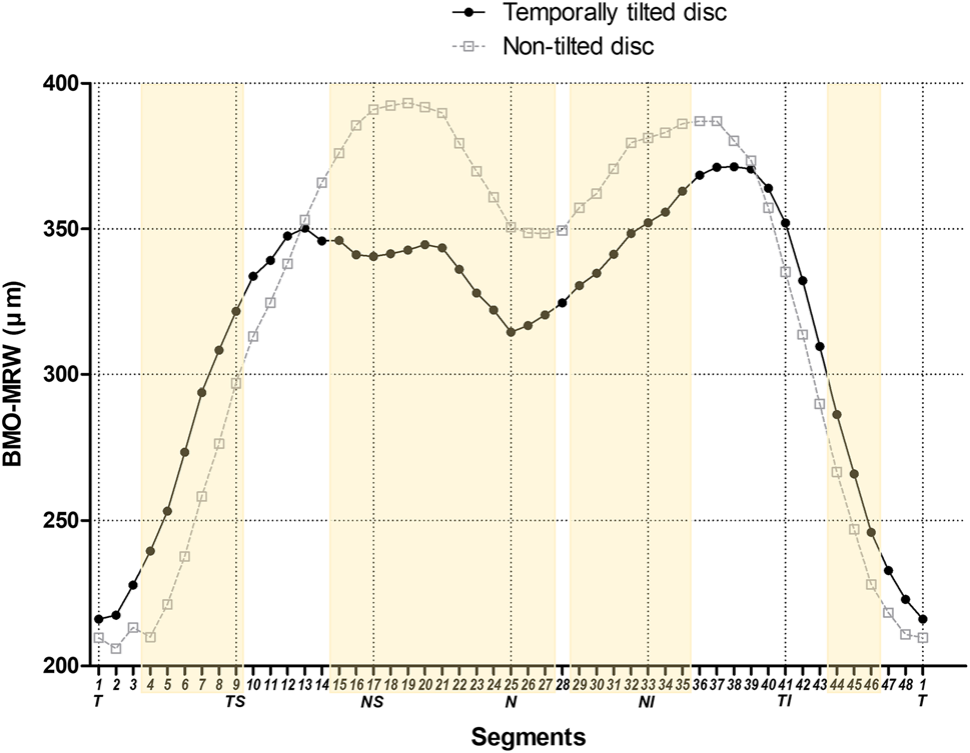
Comparison of Bruch’s membrane opening-minimum rim width in all 48 rim segments between temporally tilted and non-tilted disc groups. Abbreviations: BMO-MRW, Bruch’s membrane opening-minimum rim width; T, temporal; TS, temporal superior; NS, nasal superior; N, nasal; NI, nasal inferior; TI, temporal inferior. Segments highlighted in yellow indicate the segments with a *p*-value for comparison of the two groups based on Student’s *t*-test < 0.05.

The BMO area was larger in the temporally tilted disc group than in the non-tilted disc group (*p* < 0.001). Global BMO-MRW was similar between the two groups (*p* = 0.161). However, the temporally tilted disc group showed thicker BMO-MRW in the temporal sector (*p* = 0.032) and thinner BMO-MRW in the nasal superior and nasal sectors (*p* = 0.025 and *p* = 0.002, respectively) (Table 2, Fig. 1). The BMO-MRW was calculated for each of the 48 radial rim segments described in Fig. 2 (Supplementary Table S1, Fig. 3).

**Figure 4:**
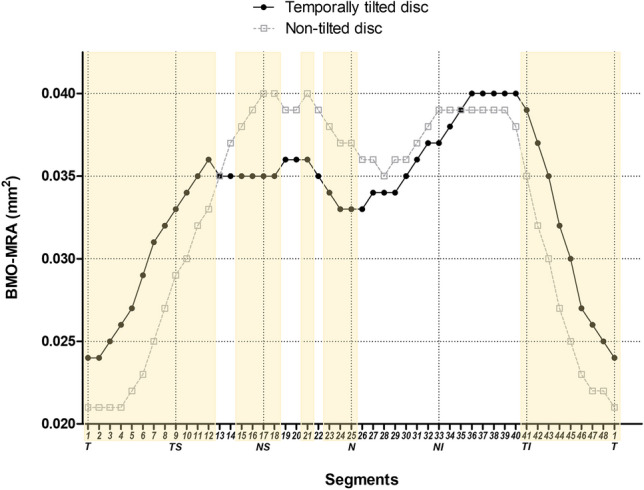
Comparison of Bruch’s membrane opening-minimum rim area in all 48 rim segments between temporally tilted and non-tilted disc groups. Abbreviations: BMO-MRA, Bruch’s membrane opening-minimum rim area; T, temporal; TS, temporal superior; NS, nasal superior; N, nasal; NI, nasal inferior; TI, temporal inferior. Segments highlighted in yellow indicate segments with a *p*-value for comparison of the two groups based on Student’s *t*-test < 0.05.

The total BMO-MRA was similar between the two groups (*p* = 0.410). However, the temporally tilted disc group showed larger BMO-MRA in the temporal, temporal superior, and temporal inferior sectors compared to the non-tilted disc group (*p* < 0.001, *p* < 0.001, and *p* = 0.016, respectively) (Table 2, Fig. 1). BMO-MRA was also calculated for each of the 48 radial rim segments described in Fig. 2 (Supplementary Table S2, Fig. 4).

**Figure 5:**
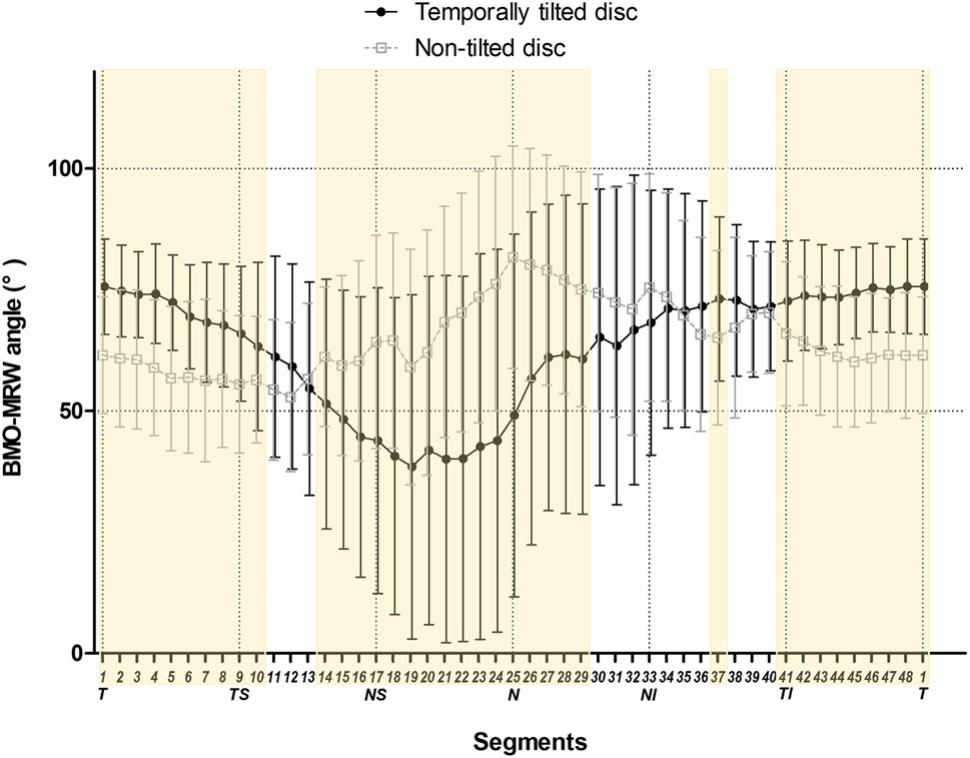
Comparison of Bruch’s membrane opening-minimum rim width angle in all 48 rim segments between temporally tilted and non-tilted disc groups. Abbreviations: BMO-MRW, Bruch’s membrane opening-minimum rim width; T, temporal; TS, temporal superior; NS, nasal superior; N, nasal; NI, nasal inferior; TI, temporal inferior. Segments highlighted in yellow indicate segments with a *p*-value for comparison of the two groups based on Student’s *t*-test < 0.05.

The temporally tilted disc group showed a larger BMO-MRW angle in the temporal sector and a smaller BMO-MRW angle in the nasal superior and nasal sectors compared to the non-tilted disc group (Supplementary Table S3, Fig. 5).

## Discussion

Since a tilted optic disc can cause structural deformation of the optic nerve, understanding the transformation of the neuroretinal rim morphology induced by the tilted optic disc is important. In addition, investigating the impact of the optic disc tilt in healthy eyes is imperative, considering the potential confounding effects of glaucoma on neuroretinal rim morphology. To the best of our knowledge, this is the first study to investigate BMO-MRW and BMO-MRA between eyes with temporally tilted and non-tilted optic discs in healthy individuals.

In our study, the spherical equivalent was lower in the temporally tilted disc group compared to the non-tilted disc group with borderline significance, while the axial length was significantly longer in the temporally tilted disc group. These findings may be attributed to the high incidence of laser refractive surgery among our study subjects. The effects of refractive surgery might have offset the difference in axial length between the two groups, resulting in no significant difference in the spherical equivalent (Table 1).

While few studies have examined the influence of myopia and optic disc tilt on BMO-based parameters, some studies have been conducted on pRNFLT in myopic tilted discs in healthy individuals^19–21,25^. Hwang et al.^21^ investigated the correlation between myopic tilted discs and characteristics of pRNFLT in healthy young men and reported that eyes with more temporally tilted discs had higher myopia, greater axial length, thinner average, superior, nasal, and inferior RNFL, and thicker temporal RNFL. In accordance, our study showed that the pRNFLT of the temporally tilted disc group was thicker in the temporal sector and thinner in the nasal superior, nasal, and nasal inferior sectors than that of the non-tilted disc group while the global pRNFLT were similar between the two groups (Table 2, Fig. 1). The mechanism of these sectoral RNFLT differences between temporally tilted and non-tilted disc eyes may involve retinal dragging towards the temporal horizon from the temporally tilted optic disc^10,21,29^.

However, Kim et al.^25^ reported that the global and sectoral pRNFLT as well as the location of RNFL peaks remained stable during myopic progression when evaluated from fixed reference circles. The study suggested that the temporalized RNFL peaks in myopic eyes in previous studies were due to the nasalized scan-circle centered on the displaced myopic ONH. Sawada et al.^30^ also stated that the pRNFLT acquired at the disc center differed from that acquired at the BMO center. Consequently, the observed discrepancy in the sectoral pRNFLT between the two groups in our study could potentially be attributed to variations in the centering of the scan-circle, which is influenced by the degree of myopia.

Since the wide usage of SD-OCT, evaluating BMO-based parameters, such as BMO-MRW, offers a more accurate evaluation of the neuroretinal rim. BMO-MRW has been well acknowledged as a more stable diagnostic marker for detecting glaucoma^31–33^. Rezapour et al.^17^ investigated 452 glaucomatous eyes with varying degrees of myopia and reported that eyes with higher myopia had greater optic disc tilt and thinner nasal superior BMO-MRW. However, since the severity of glaucoma can disguise the actual transformation of the neuroretinal rim morphology derived from the optic disc tilt, we evaluated BMO-MRW regarding optic disc tilt in healthy eyes. Our study revealed that the BMO-MRW of the temporally tilted disc group was thicker in the temporal sector and thinner in the nasal superior and nasal sectors than that of the non-tilted disc group (Table 2, Fig. 1).

BMO-MRW is a one-dimensional ONH parameter with the following limitations. Normative BMO-MRW values depend on ONH size, and in large optic discs, BMO-MRW is known to be physiologically thinner^34–36^. Therefore, matching the BMO area is necessary when comparing subjects with different sizes of BMO area to obtain an accurate value of BMO-MRW. Additionally, in highly myopic eyes, the optic disc and BMO size are known to enlarge with axial length elongation^26,37^. During myopic changes of the eye, BMO enlarges and often shifts over the optic disc in the temporal direction, and the nasal part slides into the nasal region of the optic disc^10,15^.

BMO-MRA, which is a two-dimensional ONH parameter that demonstrates a comparable level of diagnostic power to detect glaucoma to RNFLT and BMO-MRW, is known to equalize the rim width difference among various ONH sizes^34,35^. Moreover, it is reported that BMO-MRA has comparable diagnostic capability for glaucoma with the RNFLT and BMO-MRW in myopic populations and might have advantages in macrodiscs^36^. Therefore, we analyzed BMO-MRA between temporally tilted and non-tilted disc groups and aimed to obtain more accurate neuroretinal rim data for discs with different BMO sizes due to myopic tilted disc.

In our study, BMO-MRA of the temporally tilted disc group was larger in the temporal, temporal superior, and temporal inferior sectors than that of the non-tilted disc group (Table 2, Fig. 1). The trend in the difference of BMO-MRA between the two groups in the temporal sector was similar to that of RNFLT and BMO-MRW. However, in the nasal sector, the difference between the two groups seemed not as significant as that of RNFLT and BMO-MRW. Meanwhile, since there could be a detection loss of localized values which could have a negligible impact on the sectoral values, we analyzed BMO-MRA not only by the sectors, but also by dividing the rim into segments 1 to 48 (Supplementary Table S2, Fig. 4). In the comparison of the localized values, BMO-MRA was significantly smaller in many segments of the nasal superior and nasal sectors in the temporally tilted disc group than in the non-tilted disc group. As a result, a temporally tilted disc can affect not only the RNFLT and BMO-MRW of temporal and nasal sectors but also BMO-MRA of temporal and nasal sectors.

Considering that the BMO-based parameters utilize BMO center as the scanning center point, these parameters were initially expected to not exhibit temporalization in the temporally tilted disc group, as shown in the RNFLT results. In addition, based on the lamina cribrosa (LC) shifting theory as a positional change of the ONH during axial elongation and myopic change, no significant sectoral disparities in BMO-based parameters were anticipated between the two groups, considering the superior placement of the BMO plane relative to the LC plane^23^. However, contrary to these expectations, BMO-based parameters showed temporalization in the temporally tilted disc group compared to the non-tilted disc group. We presume that this is due to the temporal tilting of the optic disc during myopic changes. During temporal tilting of the optic disc, the nasal half of the optic disc elevates anteriorly, while the temporal half depresses posteriorly, resulting in thickening of the BMO-MRW, enlargement of the BMO-MRA in the temporal sector, and thinning and reduction in the nasal sector. Further studies are required regarding the temporalization of BMO-based parameters.

BMO-MRA is recognized as an indicator for detecting the changes in early stages of glaucoma and a marker that responds promptly to mechanical stress, such as intraocular pressure^38^. Although the temporally tilted and non-tilted disc groups had sectoral differences, the total BMO-MRA was not different (*p* = 0.410, Table 2). There are several possible explanations for this finding. First, the extent of the tilting observed in the temporally tilted disc group could have been relatively subtle. Consequently, the observed difference may not have been significant enough to detect variations in the total BMO-MRA. Second, temporal tilt itself may not have directly affected BMO-MRA. This means that the structural tilt alone does not cause stress or damage to the neuroretinal rim. Instead, relative changes in ONH structures induced by tilting may render them more susceptible to mechanical stress, such as intraocular pressure.

Therefore, to consider the potential region of nerve fiber stress, we calculated the angle of the BMO plane and BMO-MRW in each of the 48 rim segments, which we termed as the “BMO-MRW angle”. We hypothesized that the degree of the BMO-MRW angle could indicate the anatomical stress on the nerve fibers due to compression from the relatively rigid Bruch’s membrane. We found that the BMO-MRW angle values of the temporally tilted disc group were smaller in the nasal sector and larger in the temporal sector than those of the non-tilted disc group (Supplementary Table S3, Fig. 5). This implies that in temporally tilted disc eyes, nerve fibers in the nasal sector are subjected to greater stress than those in non-tilted disc eyes because the angle between the nerve fibers and the BMO plane is steeper. This finding may be related to previous studies on temporal visual field defects observed in myopic eyes^39–41^. Ohno-Matsu et al.^39^ investigated visual field defects in high myopia and reported that a relatively high proportion of eyes with vertically oval temporally tilted discs exhibited temporal visual field defects. The study also suggested that the protrusion of the nasal edge of the optic disc caused by myopic tilt distorts the nasal nerve fibers at the edge of the optic disc and impairs axonal flow in the nasal nerve fibers, potentially causing temporal visual field defects. This finding is consistent with our hypothesis regarding the BMO-MRW angle.

However, some studies have suggested an alternative explanation for the location of glaucomatous damage in myopic glaucoma, attributing it to the tensile stress exerted on the LC resulting from LC plane shifting^42–44^. Lee et al.^43^ stated that the central vascular trunk of the optic disc could serve as an indicator of LC shift and reported that the vascular trunk is located in the direction opposite to the RNFL defects, with reference to BMO. This indicates that individuals with a temporally tilted disc, which means a nasal shift of the LC plane, may exhibit nerve fiber damage and LC defects in the temporal superior or temporal inferior sector due to tensile stress.

Our study has some limitations. First, we used one of several methods to evaluate the ovality index. Various studies have previously demonstrated techniques for calculating and quantifying the optic disc tilt, but a standardized approach has not been established^8,45–47^. However, we do not think this is a significant issue because most methods employed for calculating the optic disc tilt utilize the same concept, which is the ratio of the longest and shortest diameters of the optic disc. Second, measurement errors when calculating the ovality index and BMO-MRA may have existed since the calculations were performed manually. Nevertheless, all measurements were based on reference points analyzed using SD-OCT; thus, deviations were anticipated to be minimal. Finally, BMO points and BMO-based parameters which are automatically detected and analyzed by the SD-OCT software, may have been miscalculated in the first place especially in tilted disc eyes. However, we manually reviewed the SD-OCT data of all subjects to prevent this type of error.

In conclusion, the neuroretinal rim, represented by BMO-MRW and BMO-MRA, showed morphological differences between temporally tilted and non-tilted optic discs in healthy eyes. BMO-MRW and BMO-MRA showed temporalization in the same manner as pRNFLT in temporally tilted disc eyes compared to non-tilted disc eyes. The angle between the BMO plane and BMO-MRW, which we termed as the “BMO-MRW angle” showed that in temporally tilted disc eyes, optic nerve fibers met the BMO plane steeply in the nasal sector and gently in the temporal sector than in non-tilted disc eyes, suggesting the potential stress region of optic nerve fibers in temporally tilted disc eyes. Further studies are required to explore its association with other morphological or functional parameters and, its potential role in the pathophysiology of glaucoma associated with myopia.

## Methods

This prospective cross-sectional study was reviewed and approved by the Institutional Review Board (IRB) of Kangdong Sacred Heart Hospital (Seoul, South Korea, IRB No. 2018-04-016). The study adhered to the tenets of the Declaration of Helsinki. Written informed consent was submitted by all study participants when they were enrolled.

### Subjects

Among the participants who visited the Department of Ophthalmology, Kangdong Sacred Heart Hospital, between June 2018 and April 2022, those who met the following inclusion and exclusion criteria were enrolled. The inclusion criteria were participants whose age was from 20 to 40 years with a temporally tilted or non-tilted optic disc. To evaluate the optic disc tilt, SD-OCT (Spectralis HRA&OCT, software version 1.10.2.0; Heidelberg Engineering, Heidelberg, Germany) examination was conducted. Optic disc tilt was assessed as described in the “Evaluation of optic disc tilt” section below. The exclusion criteria were participants diagnosed with ocular hypertension, glaucoma suspect, glaucoma, or other retinal or optic disc disorders. All examinations were performed on both eyes of each participant; when both eyes of a participant were eligible, only one eye was randomly selected for the analysis.

All participants underwent comprehensive ophthalmic examinations, including measurement of BCVA, refraction, slit-lamp examination, Goldmann applanation tonometry, central corneal thickness and axial length (OA-2000; Tomey Ltd, Japan), color disc and red-free RNFL photography (TRC-NW8 digital camera; Topcon Corp., Tokyo, Japan), wide-field fundus photography (Optos 200Tx, Marlborough, MA, USA), and automated perimetry using the 24-2 Swedish interactive threshold algorithm standard program (Humphrey Field Analyzer, HFA II; Carl Zeiss Meditec Inc., Dublin, CA). In addition, SD-OCT was performed on every participant to assess the pRNFLT and ONH parameters.

To exclude participants diagnosed with glaucoma suspect or glaucoma, color disc and red-free RNFL photography, SD-OCT, and automated perimetry were evaluated by an experienced glaucoma specialist (K.I.N.). The following cases showing glaucomatous changes were excluded from the study: characteristic glaucomatous changes in the optic disc, definite thinning, or defects in the pRNFL except for the temporalization, or glaucomatous visual field defects in automated perimetry. Glaucomatous visual field defects were defined as meeting at least two of the following three criteria with reliable automated perimetry (false-positive error < 15%, false-negative error < 15%, and fixation loss < 20%): (1) a cluster of three points with < 5% probability on the pattern deviation map in at least one hemifield, with at least one point with a probability of < 1%, (2) a glaucoma hemifield test result outside normal limits, and (3) a pattern standard deviation outside 95% of the normal limits.

### Evaluation of the optic disc tilt

SD-OCT scans centered on the optic disc and macula were obtained from each eligible eye by a single experienced examiner. The optic disc tilt was determined from the infrared image of this SD-OCT scan by an ophthalmologist (C.W.J.) who was blinded to participants’ information. The optic disc tilt was identified using the ovality index (ovality index = shortest optic disc diameter/longest optic disc diameter). A temporally tilted optic disc was defined as an optic disc with tilt axes within 30° of the vertical meridian temporally and with an ovality index < 0.80, as described in a previous study^47^. A non-tilted optic disc was defined as an optic disc with an ovality index > 0.90 (Fig. 2)^47^. Image J software (National Institutes of Health, Bethesda, MD, USA) was used to measure the optic disc diameter and calculate the ovality index.

### Evaluation of pRNFLT

pRNFLT at each of the six Garway-Heath sectors (temporal, temporal superior, nasal superior, nasal, nasal inferior, and temporal inferior) and the global sector were obtained automatically by a 3.5 mm-diameter peripapillary circle scan centered at the optic disc^48^. Improperly drawn RNFL segmentation lines were manually corrected by K.I.N., and automatic recalculation was performed with modified RNFL segments.

### Evaluation of ONH parameters

For the acquisition of ONH parameters, the scan pattern was centered on the BMO with 24 radially equidistant B-scans. Each B-scan delineated the rim area using a circular sector of 7.5°; a total of 48 rim measurements were obtained. The BMO and inner limiting membrane (ILM) were automatically segmented and confirmed by K.I.N. to exclude possible segmentation or sliding errors. Other inappropriate segmentations were corrected manually. The BMO-MRW was automatically calculated based on the shortest distance from the BMO point to the ILM within 48 rim segments from each identified B-scan. We numbered the 48 rim segments from 1 to 48, starting from the temporal segment, which is in line with the fovea-BMO center axis to the superior, nasal, and inferior regions of each eye (Fig. 2).

**Figure 6:**
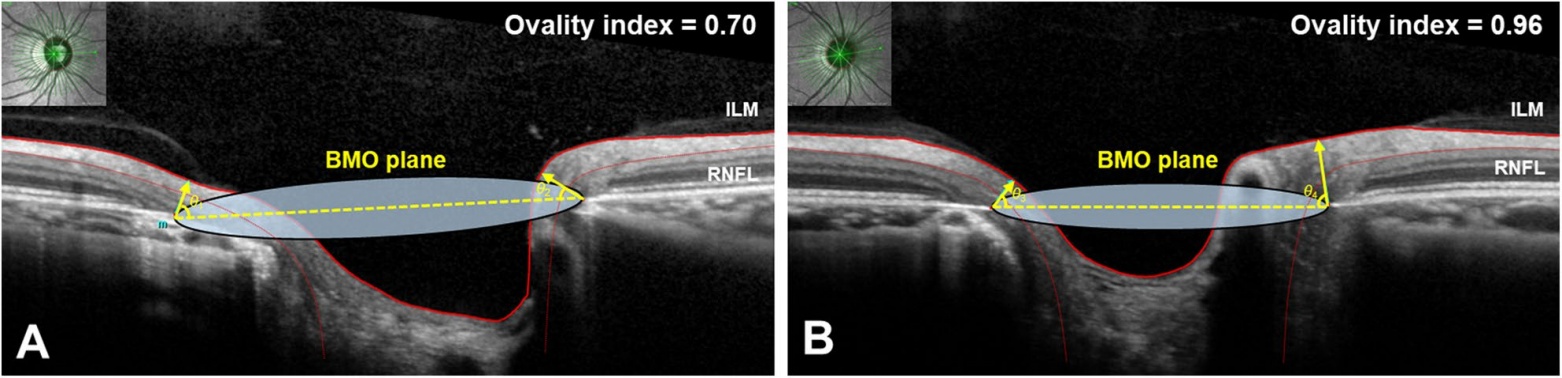
Illustrative images of the Bruch’s membrane opening-minimum rim width (BMO-MRW) angle (*θ)* and location of Bruch’s membrane opening plane in the optical coherence tomography (OCT) scan of temporally tilted and non-tilted disc eyes. (**A**) An OCT scan of the right eye from a subject in the temporally tilted disc group with an ovality index of 0.70. The temporal BMO-MRW angle (*θ*_1_*)* is 65.93°. The nasal BMO-MRW angle (*θ*_2_) is 33.76°. (**B**) An OCT scan of the right eye from a subject in the non-tilted disc group with an ovality index of 0.96. The temporal BMO-MRW angle (*θ*_3_*)* is 50.95°. The nasal BMO-MRW angle (*θ*_4_) is 82.07°. Abbreviations: BMO, Bruch’s membrane opening; ILM, internal limiting membrane; RNFL, retinal nerve fiber layer.

Because the BMO-MRW is located at an angle *θ* to the BMO plane that differs among the 48 rim segments, we calculated each of the 48 angle *θ*. We named angle *θ* as the term “BMO-MRW angle” (Fig. 6).

The BMO-MRA was calculated for each area of the 48 trapeziums in the minimum surface area between the BMO and ILM at an angle *θ* above the BMO plane. The height of the trapezium was set to be equal to the rim width (*W*) at this angle. The base of the trapezium was measured by the circumference length of the BMO in each sector, which is, 2π*r*/48, where *r* was defined as the length from the BMO centroid to each BMO point. The top of the trapezium was measured using the following formula: 2π/48 × (*r* − *W* × cos*θ*). The area of each trapezium was calculated using the following formula: Area = (2π*r*/48 + 2π/48 × [*r* − *W* × cos*θ]*)/2 × *W*^28^. The length from the BMO centroid to each 48 BMO point (*r*) and the angle *θ* were measured manually using Image J software.

The delineated 48 circular areas were grouped into six sectors: temporal sector of 90°, temporal superior sector of 37.5°, nasal superior sector of 37.5°, nasal sector of 120°, nasal inferior sector of 37.5°, and temporal inferior sector of 37.5°.

### Statistical analyses

All statistical analyses were conducted using SPSS software for Windows (version 27.0; SPSS Inc., Chicago, Illinois, USA). Snellen BCVA results were converted into the logarithm of the minimum angle of resolution value. Student’s *t*-test or χ^2^ test was used to assess the differences between the temporally tilted and non-tilted disc groups. Continuous variables are presented as mean ± standard deviation. In all analyses, differences were considered significant at a *p*-value < 0.05.

### Supplementary Information

Supplementary Tables.

## Data Availability

The datasets generated during the current study are available from the corresponding author upon request.
