# Supplementary material for: Morphological differences of the neuroretinal rim between temporally tilted and non-tilted optic discs in healthy eyes
Source: Sci Rep. 2024 Mar 13;14:6070. doi: 10.1038/s41598-024-54116-7 (PMC10937920; doi:10.1038/s41598-024-54116-7)
Supplement: Supplementary file 1 — Supplementary Tables. [file 41598_2024_54116_MOESM1_ESM.pdf]

Morphological Differences of the Neuroretinal Rim between Temporally Tilted and Non-tilted Optic Discs in Healthy Eyes

Supplementary Table S1. Comparison of Bruch's membrane opening-minimum rim width in all 48 rim segments between the temporally tilted and non-tilted disc groups

	Temporally tilted disc group (n=55)	Non-tilted disc group (n=38)	*p*-value
BMO-MRW 1	216.15 ± 38.43	209.66 ± 35.91	0.413
BMO-MRW 2	217.51 ± 40.56	206.00 ± 32.06	0.147
BMO-MRW 3	227.80 ± 44.75	213.26 ± 53.26	0.158
BMO-MRW 4	239.53 ± 48.71	209.97 ± 35.04	0.002
BMO-MRW 5	253.22 ± 52.40	221.11 ± 33.44	0.001
BMO-MRW 6	273.44 ± 51.82	237.66 ± 33.12	< 0.001
BMO-MRW 7	293.96 ± 55.37	258.24 ± 36.97	0.001
BMO-MRW 8	308.35 ± 54.13	276.37 ± 39.50	0.002
BMO-MRW 9	321.71 ± 55.67	297.08 ± 41.78	0.023
BMO-MRW 10	333.73 ± 51.88	312.97 ± 46.23	0.051
BMO-MRW 11	339.20 ± 56.79	324.71 ± 45.20	0.193
BMO-MRW 12	347.56 ± 59.55	338.05 ± 50.98	0.425
BMO-MRW 13	350.27 ± 59.04	353.08 ± 54.05	0.816
BMO-MRW 14	345.85 ± 63.57	365.87 ± 51.62	0.111
BMO-MRW 15	345.98 ± 65.23	376.03 ± 47.08	0.017
BMO-MRW 16	341.09 ± 68.68	385.55 ± 51.04	0.001
BMO-MRW 17	340.49 ± 76.76	390.95 ± 56.96	0.000
BMO-MRW 18	341.47 ± 81.87	392.37 ± 65.84	0.001
BMO-MRW 19	342.69 ± 82.45	393.26 ± 68.39	0.002

BMO-MRW 20	344.58 ± 82.68	391.87 ± 67.99	0.005
BMO-MRW 21	343.47 ± 83.78	389.79 ± 66.71	0.006
BMO-MRW 22	336.09 ± 88.73	379.50 ± 64.76	0.012
BMO-MRW 23	328.02 ± 85.41	369.82 ± 60.49	0.007
BMO-MRW 24	322.15 ± 81.70	360.84 ± 60.04	0.010
BMO-MRW 25	314.51 ± 80.45	350.58 ± 56.73	0.019
BMO-MRW 26	316.80 ± 76.55	348.68 ± 54.94	0.022
BMO-MRW 27	320.49 ± 69.63	348.37 ± 55.31	0.042
BMO-MRW 28	324.56 ± 64.03	349.39 ± 57.08	0.058
BMO-MRW 29	330.51 ± 63.62	357.29 ± 54.25	0.037
BMO-MRW 30	334.80 ± 67.76	362.11 ± 55.54	0.043
BMO-MRW 31	341.27 ± 68.15	370.63 ± 50.74	0.026
BMO-MRW 32	348.42 ± 68.82	379.58 ± 52.43	0.021
BMO-MRW 33	352.11 ± 70.54	381.21 ± 47.17	0.019
BMO-MRW 34	355.75 ± 68.82	383.05 ± 42.31	0.020
BMO-MRW 35	362.96 ± 64.26	386.11 ± 41.04	0.037
BMO-MRW 36	368.42 ± 64.35	386.97 ± 42.81	0.098
BMO-MRW 37	371.18 ± 61.63	386.97 ± 44.97	0.180
BMO-MRW 38	371.35 ± 62.05	380.24 ± 47.67	0.459
BMO-MRW 39	370.56 ± 64.21	373.47 ± 46.11	0.800
BMO-MRW 40	363.96 ± 63.79	357.32 ± 44.74	0.556
BMO-MRW 41	352.04 ± 64.60	335.29 ± 43.44	0.138
BMO-MRW 42	332.20 ± 60.87	313.68 ± 43.40	0.110
BMO-MRW 43	309.62 ± 58.49	290.05 ± 39.52	0.057
BMO-MRW 44	286.44 ± 53.57	266.71 ± 36.60	0.038
BMO-MRW 45	265.98 ± 44.96	246.92 ± 36.78	0.033
BMO-MRW 46	245.93 ± 39.28	228.11 ± 31.81	0.018

BMO-MRW 47	232.82 ± 40.31	218.32 ± 29.91	0.062
BMO-MRW 48	222.91 ± 39.67	210.87 ± 31.13	0.121

Values are presented as mean ± standard deviation unless otherwise indicated.

Abbreviations: BMO-MRW 1-48, Bruch's membrane opening-minimum rim width calculated at each segment from segment 1, which is aligned parallel to the fovea-Bruch's membrane opening axis to segment 48 clockwise in the right eye, and counterclockwise in the left eye.

P-values were calculated using the Student's *t*-test for all variables.

Supplementary Table S2. Comparison of Bruch's membrane opening-minimum rim area in all 48 rim segments between the temporally tilted and non-tilted disc groups

	Temporally tilted disc group (n=55)	Non-tilted disc group (n=38)	*p*-value
BMO-MRA 1	0.024 ± 0.004	0.021 ± 0.005	0.012
BMO-MRA 2	0.024 ± 0.004	0.021 ± 0.004	0.001
BMO-MRA 3	0.025 ± 0.004	0.021 ± 0.005	0.001
BMO-MRA 4	0.026 ± 0.005	0.021 ± 0.004	< 0.001
BMO-MRA 5	0.027 ± 0.005	0.022 ± 0.004	< 0.001
BMO-MRA 6	0.029 ± 0.005	0.023 ± 0.004	< 0.001
BMO-MRA 7	0.031 ± 0.005	0.025 ± 0.004	< 0.001
BMO-MRA 8	0.032 ± 0.005	0.027 ± 0.004	< 0.001
BMO-MRA 9	0.033 ± 0.005	0.029 ± 0.004	< 0.001
BMO-MRA 10	0.034 ± 0.005	0.030 ± 0.004	< 0.001
BMO-MRA 11	0.035 ± 0.005	0.032 ± 0.004	0.003
BMO-MRA 12	0.036 ± 0.006	0.033 ± 0.005	0.017
BMO-MRA 13	0.035 ± 0.006	0.035 ± 0.005	0.615
BMO-MRA 14	0.035 ± 0.007	0.037 ± 0.005	0.234
BMO-MRA 15	0.035 ± 0.007	0.038 ± 0.005	0.027
BMO-MRA 16	0.035 ± 0.007	0.039 ± 0.005	0.003
BMO-MRA 17	0.035 ± 0.008	0.040 ± 0.006	0.007
BMO-MRA 18	0.035 ± 0.009	0.040 ± 0.007	0.021
BMO-MRA 19	0.036 ± 0.011	0.039 ± 0.007	0.125
BMO-MRA 20	0.036 ± 0.011	0.039 ± 0.008	0.145
BMO-MRA 21	0.036 ± 0.010	0.040 ± 0.007	0.042

BMO-MRA 22	0.035 ± 0.010	0.039 ± 0.008	0.051
BMO-MRA 23	0.034 ± 0.010	0.038 ± 0.007	0.040
BMO-MRA 24	0.033 ± 0.010	0.037 ± 0.006	0.035
BMO-MRA 25	0.033 ± 0.009	0.037 ± 0.007	0.020
BMO-MRA 26	0.033 ± 0.009	0.036 ± 0.007	0.092
BMO-MRA 27	0.034 ± 0.008	0.036 ± 0.006	0.220
BMO-MRA 28	0.034 ± 0.008	0.035 ± 0.006	0.486
BMO-MRA 29	0.034 ± 0.007	0.036 ± 0.005	0.297
BMO-MRA 30	0.035 ± 0.008	0.036 ± 0.006	0.486
BMO-MRA 31	0.036 ± 0.009	0.037 ± 0.005	0.543
BMO-MRA 32	0.037 ± 0.009	0.038 ± 0.006	0.636
BMO-MRA 33	0.037 ± 0.009	0.039 ± 0.006	0.364
BMO-MRA 34	0.038 ± 0.009	0.039 ± 0.005	0.546
BMO-MRA 35	0.039 ± 0.008	0.039 ± 0.005	0.784
BMO-MRA 36	0.040 ± 0.008	0.039 ± 0.006	0.687
BMO-MRA 37	0.040 ± 0.008	0.039 ± 0.006	0.548
BMO-MRA 38	0.040 ± 0.008	0.039 ± 0.006	0.331
BMO-MRA 39	0.040 ± 0.008	0.039 ± 0.006	0.497
BMO-MRA 40	0.040 ± 0.008	0.038 ± 0.005	0.134
BMO-MRA 41	0.039 ± 0.009	0.035 ± 0.005	0.002
BMO-MRA 42	0.037 ± 0.008	0.032 ± 0.004	< 0.001
BMO-MRA 43	0.035 ± 0.008	0.030 ± 0.004	< 0.001
BMO-MRA 44	0.032 ± 0.007	0.027 ± 0.004	< 0.001
BMO-MRA 45	0.030 ± 0.005	0.025 ± 0.005	< 0.001
BMO-MRA 46	0.027 ± 0.005	0.023 ± 0.004	< 0.001
BMO-MRA 47	0.026 ± 0.005	0.022 ± 0.004	< 0.001
BMO-MRA 48	0.025 ± 0.005	0.022 ± 0.005	0.001

Values are presented as mean ± standard deviation unless otherwise indicated.

Abbreviations: BMO-MRA 1-48, Bruch's membrane opening-minimum rim area calculated at each segment from segment 1, which is aligned parallel to the fovea-Bruch's membrane opening axis to segment 48 clockwise in the right eye, and counterclockwise in the left eye.

P-values were calculated using the Student's *t*-test for all variables.

Supplementary Table S3. Comparison of Bruch's membrane opening-minimum rim width angle in all 48 rim segments between the temporally tilted and non-tilted disc groups

	Temporally tilted disc group (n=55)	Non-tilted disc group (n=38)	*p*-value
BMO-MRW angle 1	75.65 ± 9.82	61.52 ± 12.04	< 0.001
BMO-MRW angle 2	74.74 ± 9.44	60.83 ± 14.18	< 0.001
BMO-MRW angle 3	74.00 ± 8.90	60.60 ± 14.34	< 0.001
BMO-MRW angle 4	74.14 ± 10.27	58.91 ± 14.03	< 0.001
BMO-MRW angle 5	72.35 ± 9.84	56.73 ± 14.88	< 0.001
BMO-MRW angle 6	69.42 ± 10.72	56.92 ± 15.58	< 0.001
BMO-MRW angle 7	68.28 ± 12.36	56.28 ± 16.74	< 0.001
BMO-MRW angle 8	67.62 ± 12.66	56.55 ± 14.13	< 0.001
BMO-MRW angle 9	65.93 ± 13.86	55.49 ± 14.21	0.001
BMO-MRW angle 10	63.26 ± 17.37	56.43 ± 13.07	0.043
BMO-MRW angle 11	61.16 ± 20.74	54.36 ± 14.50	0.084
BMO-MRW angle 12	59.16 ± 21.16	52.84 ± 15.35	0.118
BMO-MRW angle 13	54.63 ± 22.02	56.58 ± 15.59	0.640
BMO-MRW angle 14	51.42 ± 25.78	61.18 ± 14.42	0.022
BMO-MRW angle 15	48.25 ± 26.67	59.34 ± 18.56	0.020
BMO-MRW angle 16	44.65 ± 28.95	60.32 ± 20.63	0.003
BMO-MRW angle 17	43.87 ± 31.56	64.19 ± 22.00	< 0.001
BMO-MRW angle 18	40.69 ± 32.71	64.48 ± 22.20	< 0.001
BMO-MRW angle 19	38.48 ± 35.50	59.06 ± 24.30	0.001
BMO-MRW angle 20	41.88 ± 35.94	62.05 ± 25.32	0.002
BMO-MRW angle 21	40.08 ± 37.89	68.37 ± 23.83	< 0.001
BMO-MRW angle 22	40.15 ± 37.66	70.29 ± 24.64	< 0.001
BMO-MRW angle 23	42.65 ± 39.76	73.53 ± 25.95	< 0.001

BMO-MRW angle 24	43.87 ± 39.51	76.26 ± 26.26	< 0.001
BMO-MRW angle 25	49.08 ± 37.41	81.73 ± 22.95	< 0.001
BMO-MRW angle 26	56.68 ± 34.34	80.21 ± 23.96	< 0.001
BMO-MRW angle 27	61.06 ± 31.61	79.02 ± 23.72	0.002
BMO-MRW angle 28	61.66 ± 32.80	77.01 ± 23.49	0.010
BMO-MRW angle 29	60.70 ± 31.99	75.07 ± 24.22	0.016
BMO-MRW angle 30	65.20 ± 30.60	74.36 ± 24.44	0.113
BMO-MRW angle 31	63.44 ± 32.80	72.44 ± 23.71	0.128
BMO-MRW angle 32	66.72 ± 31.92	70.98 ± 25.98	0.481
BMO-MRW angle 33	68.19 ± 27.31	75.48 ± 23.41	0.184
BMO-MRW angle 34	71.14 ± 24.66	73.53 ± 21.48	0.630
BMO-MRW angle 35	70.72 ± 24.10	69.71 ± 19.56	0.831
BMO-MRW angle 36	71.59 ± 21.78	65.78 ± 20.06	0.195
BMO-MRW angle 37	73.11 ± 16.93	65.13 ± 18.01	0.032
BMO-MRW angle 38	72.81 ± 15.62	67.19 ± 18.64	0.118
BMO-MRW angle 39	70.98 ± 13.99	70.01 ± 12.02	0.728
BMO-MRW angle 40	71.57 ± 13.28	70.28 ± 12.55	0.638
BMO-MRW angle 41	72.67 ± 12.41	65.99 ± 14.89	0.021
BMO-MRW angle 42	73.81 ± 11.36	64.42 ± 13.26	< 0.001
BMO-MRW angle 43	73.52 ± 10.72	62.43 ± 13.25	< 0.001
BMO-MRW angle 44	73.42 ± 9.77	61.21 ± 14.50	< 0.001
BMO-MRW angle 45	74.32 ± 9.43	60.11 ± 13.45	< 0.001
BMO-MRW angle 46	75.42 ± 9.12	60.88 ± 13.32	< 0.001
BMO-MRW angle 47	75.03 ± 8.83	61.59 ± 11.77	< 0.001
BMO-MRW angle 48	75.70 ± 9.77	61.43 ± 13.00	< 0.001

Values are presented as mean ± standard deviation unless otherwise indicated.

Abbreviations: BMO-MRW angle 1-48, Bruch’s membrane opening-minimum rim width angle calculated at each

segment from segment 1, which is aligned parallel to the fovea-Bruch's membrane opening axis to segment 48 clockwise in the right eye, and counterclockwise in the left eye.

P-values were calculated using the Student's *t*-test for all variables.
